# Unpacking organizational readiness for change: an updated systematic review and content analysis of assessments

**DOI:** 10.1186/s12913-020-4926-z

**Published:** 2020-02-11

**Authors:** Isomi M. Miake-Lye, Deborah M. Delevan, David A. Ganz, Brian S. Mittman, Erin P. Finley

**Affiliations:** 1 VA Greater Los Angeles Healthcare System, Los Angeles, CA USA; 2 University of California, Los Angeles, Los Angeles, CA USA; 3 Kaiser Permanente Research, Pasadena, CA USA; 4 South Texas Veterans Health Care System, San Antonio, TX USA; 5 The University of Texas Health Science Center at San Antonio, San Antonio, TX USA

**Keywords:** Systematic review, Organizational readiness for change, Content analysis, Implementation research, Consolidated framework for implementation research

## Abstract

**Background:**

Organizational readiness assessments have a history of being developed as important support tools for successful implementation. However, it remains unclear how best to operationalize readiness across varied projects or settings. We conducted a synthesis and content analysis of published readiness instruments to compare how investigators have operationalized the concept of organizational readiness for change.

**Methods:**

We identified readiness assessments using a systematic review and update search. We mapped individual assessment items to the Consolidated Framework for Implementation Research (CFIR), which identifies five domains affecting implementation (outer setting, inner setting, intervention characteristics, characteristics of individuals, and implementation process) and multiple constructs within each domain.

**Results:**

Of 1370 survey items, 897 (68%) mapped to the CFIR domain of inner setting, most commonly related to constructs of readiness for implementation (*n* = 220); networks and communication (*n* = 207); implementation climate (*n* = 204); structural characteristics (*n* = 139); and culture (*n* = 93). Two hundred forty-two items (18%) mapped to characteristics of individuals (mainly other personal attributes [*n* = 157] and self-efficacy [*n* = 52]); 80 (6%) mapped to outer setting; 51 (4%) mapped to implementation process; 40 (3%) mapped to intervention characteristics; and 60 (4%) did not map to CFIR constructs. Instruments were typically tailored to specific interventions or contexts.

**Discussion:**

Available readiness instruments predominantly focus on contextual factors within the organization and characteristics of individuals, but the specificity of most assessment items suggests a need to tailor items to the specific scenario in which an assessment is fielded. Readiness assessments must bridge the gap between measuring a theoretical construct and factors of importance to a particular implementation.

## Background

The rapid growth of multi-disciplinary fields, including implementation science, brings along with it the propagation of more terminology [1, 2]. While some of these terms may represent unique ideas, there are also many examples of the Jingle and Jangle Fallacies [3, 4]. The Jingle Fallacy, also known as synonymy, occurs when multiple names are used to refer to the same concept or thing (e.g., practice facilitation and coaching). Conversely, the Jangle Fallacy, or polysemy, occurs when the same name is used for different concepts or things. For instance, a “practice” in healthcare could refer to a medical organization (e.g., there are three doctors at this practice) or a strategy or process (e.g., a care management practice to manage chronic illness).

The seemingly self-explanatory concept of “organizational readiness for change” actually falls prey to both the Jingle and Jangle Fallacies. In the former case, we do not yet have good distinctions between assessing “organizational readiness for change,” “needs,” “barriers and facilitators,” or “factors affecting implementation” [5]. An earlier systematic review on organizational readiness for change found that relevant literature, in addition to discussing “readiness”, used terms like “preparedness”, “willingness”, “commitment” and “acceptance” [6].

The Jangle Fallacy also applies in that “organizational readiness for change” has been defined and measured in different ways. Some definitions and measures focus on the characteristics of individuals within an organization, as demonstrated by this definition from Weiner and colleagues: “the extent to which organizational members are psychologically and behaviorally prepared to implement organizational change” [7]. Others focus on macro-level factors, such as collective commitment or collective efficacy, and define organizational readiness for change as “a comprehensive attitude” that incorporates factors at an organizational level [8].

In the absence of a consensus on a conceptual framework for organizational readiness for change, knowing what needs to be included in such an assessment may remain a challenge [9]. Theorists in implementation science have an interest in refining and standardizing the measurement of organizational readiness for change to improve conceptual clarity, comparison across sites and studies, and predictive validity. In practice, however, using an existing measure may be challenging. Some assessments are developed with a particular setting or intervention in mind [6], for example, specific to addiction treatment [10], or describing transitions related to a hospital relocation [11] which can makes them less generalizable. On the other hand, broader assessments, in their attempts to be inclusive, may be lengthy or imprecise and thus require adaptation to meet the needs of a given context.

Our work began as part of the US Department of Veterans Affairs Health Services Research and Development (HSR&D) Care Coordination Quality Enhancement Research Initiative (QUERI) program. One of our aims was to use readiness assessments across three different projects to improve care coordination in VA and compare their predictive validity regarding implementation outcomes. We began by searching for existing assessments and discovered that a team at St. Michael’s Hospital in Toronto had created the Ready, Set, Change! decision support tool to help researchers identify existing assessments that would be best suited for their studies [12]. The Ready, Set, Change! team included assessments from a 2014 systematic review [6] that met pre-determined criteria for validity and reliability. The recommended assessments from the decision support tool, however, were not suitable for our needs without adaptation, due to their length and lack of relevance to our specific context and intervention details.

In response to this experience, we set out to review existing measures of organizational readiness for change to see how others had operationalized the concept. We then engaged in content analysis to identify core concepts, mapping them to the Consolidated Framework for Implementation Research (CFIR) [13]. CFIR provides a broad range of constructs relevant to implementation research and allowed for comprehensive description and comparison of the explicit and implicit definitions and frameworks underlying identified readiness assessments. Because we anticipated a range of organizational readiness definitions and measurement approaches, we chose CFIR as a broad framework that would likely capture the various permutations organizational readiness assessments were likely to take, even when they did not overlap with each other or any one organizational readiness for change framework. In so building on prior work [6, 7, 12, 14], our objective is pragmatic: to support developers of readiness assessments in determining key topics they may want to keep in mind when tailoring or developing an assessment outside the purview of existing assessments.

## Methods

Our approach involved multiple steps. First, we used systematic review methods to update the database searches conducted by a prior review of organizational readiness for change assessments to identify any additional relevant assessments. Then, we built an item bank composed of individual items included in the readiness assessments identified. Finally, we used directed content analysis to sort items into categories using CFIR as our initial foundation [13]. This systematic review is reported according to Preferred Reporting Items for Systematic Reviews and Meta-Analyses (PRISMA) guidelines, see Additional file 1 for PRISMA checklist [15].

### Literature search

We built upon the literature search conducted by Gagnon and colleagues as part of their 2014 systematic review of organizational readiness instruments [6]. Because this review of organizational readiness assessments used a search conducted in 2012, we updated the search through June 14, 2017. This broad search was based on terms related to readiness, change, and health or social services within six databases: Web of Science, Sociological Abstracts, PubMed, PsycINFO, Embase, and CINAHL (see Additional file 2 for full search strategy). We found additional studies by mining identified literature for relevant references, as well as by expert suggestion.

### Study selection

Two team members (IML, DMD) independently screened all identified titles and abstracts in duplicate. For potentially relevant abstracts, we retrieved full-text articles and reviewed them independently in duplicate as well, with discrepancies reviewed by the full team. To be included, the actual assessment used, with a full list of individual items, needed to be available for each full-text publication. This assessment needed to be relevant to healthcare delivery settings and to measure organizational readiness for change. Because, as noted above, organizational readiness for change is a nebulous concept, the measure had to capture a general sentiment of willingness, readiness, or acceptance for an organizational or collective change or innovation (rather than personal behavior change, e.g., for smoking cessation). Multiple studies using the same assessment could be included if they represented unique data collection with separate samples of participants, since each use constituted an operationalization that could inform our research objective. By including duplications and variations, we were better able to describe the uses of each assessment, including contexts in which each assessment was used, if the assessment was altered, and whether assessments were collected alongside additional measures.

### Data abstraction

We transcribed all individual questions or items from included publications into a database that served as an item bank. We captured information about each included publication, including the name of the assessment used (when reported), total number of items in that assessment or assessments, study setting, study sample, type of intervention, and any additional data collected for the study (e.g., other screeners or surveys, interviews, patient records). For items that appeared multiple times, we made separate entries in the database for each unique appearance (i.e., when one assessment was used by multiple studies in part or in whole). We did not conduct a quality assessment of the included studies, since our analysis was not focused on the validity or robustness of study findings.

### Synthesis and analysis

We used directed content analysis to identify themes within the readiness assessment items in our database. Directed content analysis builds from existing theory, models, or frameworks, which can provide the initial coding structure [16]. Beginning with these predetermined codes, all data is coded to the extent possible. Analysts then identify data that cannot be captured by the existing coding structure and develop new codes, or sub-codes of existing codes, to better capture how the existing theory, model, or framework is supported and extended by the data.

Because of the conceptual fuzziness surrounding organizational readiness for change, we sought a comprehensive framework to which we could map items in the item bank, and selected CFIR, which includes five domains within which 39 constructs are nested [13]. The “intervention characteristics” domain includes eight constructs such as relative advantage and cost of the intervention. The “outer setting” domain includes four constructs for factors outside an organization (e.g., external policy and incentives). Within the “inner setting” domain are five constructs: structural characteristics, networks and communications, culture, implementation climate, and readiness for implementation. These last two constructs are also broken down into sub-constructs, with six sub-constructs nested under implementation climate and three under readiness for implementation. The fourth domain is “characteristics of individuals,” which houses five constructs. The final domain of “process” is comprised of four constructs: planning, engaging (which has sub-constructs for four different groups of individuals who may be involved in the implementation), executing, and reflecting and evaluating. For a delineation of how the framework was applied in this analysis, and exemplar items from the item bank, see the codebook in Additional file 3. We iteratively developed the codebook based on the existing framework to clarify our application of the CFIR construct definitions and any modifications we made. For example, based on the CFIR definitions, we limited certain CFIR constructs to intervention-specific items (e.g., the construct “available resources” was used for project-specific resources), whereas other CFIR constructs were exclusively used for items that described general characteristics (e.g., the construct of “structural characteristics” was applied for items describing organizational resources more broadly).

Two members of the study team independently coded each item with a CFIR construct, or sub-construct where possible. All discrepancies were reconciled by these two members or the larger team when necessary. We categorized nearly all items under a CFIR construct or sub-construct. We developed one new construct-level code to capture items related to leadership qualities that were not intervention-specific. These items did not fit into the CFIR categorizations, as the existing representation of leadership within CFIR was in sub-constructs related to engagement of leadership with a specific intervention, as opposed to a more general description of an organization’s leaders. Some additional items that were project-specific were excluded from coding (e.g., “12-step theory (AA/NA) is followed by many of the counselors here” [17]). When more than 50 items were coded to a CFIR construct that did not have specific sub-constructs, a pile- sort methodology was used to develop new sub-constructs; this allowed us to better characterize the diversity within these large constructs.

In the case of the networks and communications construct, we used an additional model from Lanham and colleagues to classify the sub-constructs, since emerging sub-codes aligned with characteristics of work relationships that Lanham and colleagues had previously identified [18–20]. CFIR defines the networks and communications construct as being about relationships: “the nature and quality of webs of social networks and the nature and quality of formal and informal communications within an organization” [13]. Specifying sub-constructs using an established model for work relationships therefore had face validity.

The Lanham model was developed with a focus on relationships in healthcare delivery settings; applications of the model suggest that these relationship characteristics should be considered during improvement efforts or redesign [19, 20]. The model includes seven characteristics, of which five emerged within these data and were therefore applied: relatedness, trust, respectful interaction, heedfulness, and mindfulness. Full descriptions of these five characteristics are provided in Additional file 3. We generated additional inductive sub-constructs to capture emergent themes in the items within the networks and communications construct that fell outside the relationship model.

In coding each item, we relied on the most granular code appropriate (e.g., using subcodes where appropriate), and noted the unit of measurement: “self,” “staff,” “leadership,” or “organization.” “Organization” was the default if the unit of measurement was ambiguous. Additionally, we recorded information on whether the item referenced implementation of a specific intervention, rather than a general question about the state of the organization or individual. See Additional file 4 for the coding form. Once all items were coded, we narratively summarized our findings to describe the operationalization of organizational readiness for change within the included assessments and studies.

## Results

### Literature flow

**Fig. 1:**
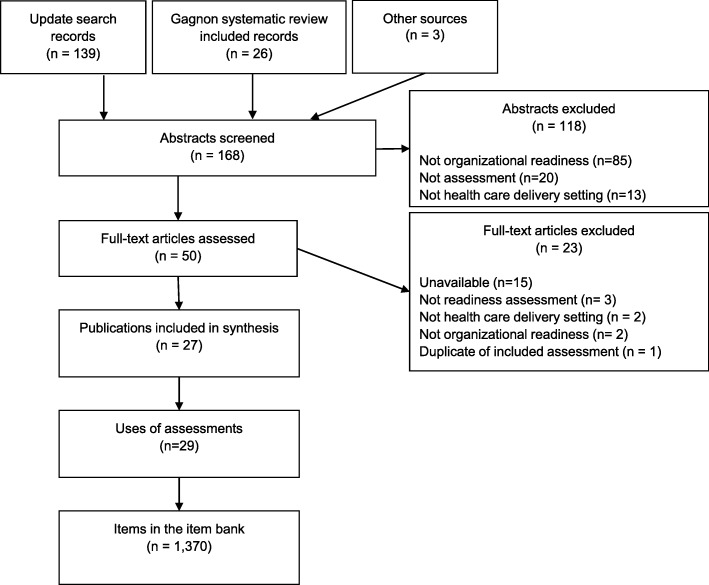
Literature Flow

The total number of publications included in our analysis is 27, which represents 29 uses of readiness assessments. From the 29 of organizational readiness assessment uses, 1370 individual assessment items were included in the item bank. See Fig. 1 for literature flow.

### Description of included studies

Table 1 provides details of the published uses of readiness assessments. The first instance was in 1988 [37], with the next one a decade later [29]. Published uses of readiness assessments steadily increased from 2007 onward, with 23 of the 29 assessment uses published in 2007 or later. Readiness assessment data were supplemented with additional data collection efforts in 14 uses, which commonly took the form of interviews or other complementary instruments, such as a job satisfaction survey [22].

**Table 1:** Evidence Table for Included Studies

Study	Assessment	Study description
ORC-BASED ASSESSMENTS		
Lehman, 2002a [10]	Assessment name: Organizational Readiness for Change Treatment Director Version (ORC-D) Items mapped/items total: 104/116	Setting: Addiction Technology Transfer centers Sample: 135 directors from 101 treatment units Type of intervention: N/A Additional data in study: A program identification form (PID) and a patient-level form (the CEST)
Lehman, 2002b [10]	Assessment name: Organizational Readiness for Change Treatment Staff Version (ORC-S) Items mapped/items total: 104/116	Setting: Addiction Technology Transfer centers Sample: 458 treatment personnel from 111 treatment units Type of intervention: N/A Additional data in study: A program identification form (PID) and a patient-level form (the CEST)
Bohman, 2008 [21]	Assessment name: Medical Organizational Readiness for Change survey Items mapped/items total: 44/45	Setting: Trauma center (community health programs) Sample: 141 Community Health Program (CHP) and 45 Emergency Center (EC) respondents Type of intervention: A screening, brief intervention, and referral to treatment (SBIRT) program for alcohol and drug misuse Additional data in study: None
Claiborne, 2013 [22]	Assessment name: N/A Items mapped/items total: 9/9	Setting: Not-for-profit child welfare agencies under contract with the public child welfare system in one state participating in the Children’s Bureau supported child welfare workforce project Sample: 356 direct care and clinical child welfare workers Type of intervention: N/A Additional data in study: The Spector Job Satisfaction Survey, Parker Organizational Climate survey
Guerrero, 2016 [17]	Assessment name: Modified version of ORC-D Items mapped/items total: 79/84	Setting: Publicly funded substance abuse treatment organizations Sample: 97 programs Type of intervention: Evaluated program capacity factors associated with client outcomes Additional data in study: Clinical encounter data from the Los Angeles County Participant Reporting System (LACPRS), leadership scale, Cultural Competence Self-Assessment Questionnaire
Saldana, 2007 [23]	Assessment name: Organizational Readiness for Change Treatment Staff Version (ORC-S) Items mapped/items total: 124/134	Setting: State mental health and substance abuse treatment sectors Sample: 543 community-based therapists treating substance-abusing youth Type of intervention: N/A Additional data in study: Personnel Data Inventory, EBPAS, a modified version of a questionnaire to evaluate staff attitudes toward treatment manuals.
ASSESSMENTS BASED ON HOLT, 2007		
Holt, 2007 [8]	Assessment name: Readiness for Organizational Change scale Items mapped/items total: 41/41	Setting: Government organization that was responsible for developing and fielding information systems for the Department of Defense Sample: 264 employees Type of intervention: New organizational structure being implemented Additional data in study: None
Saleh, 2016 [24]	Assessment name: Adapted Readiness for Organizational Change scale Items mapped/items total: 41/41	Setting: Primary Health Care centers in Lebanon Sample: 213 primary healthcare providers (physicians, nurses, other providers) working in 22 PHC centers Type of intervention: eHealth tools Additional data in study: None
EVIDENCE-BASED PRACTICES (EBP) BELIEFS SCALE		
Melnyk, 2008 [25]	Assessment name: Evidence-Based Practices (EBP) Beliefs scale Items mapped/items total: 16/16	Setting: Nurses from five states in the U.S. who attended continuing education workshops on EBP Sample: 394 nurses Type of intervention: EBP Additional data in study: EBP implementation scale
Breckenridge-Sproat, 2015 [26]	Assessment name: Evidence-Based Practices (EBP) Beliefs scale Items mapped/items total: 16/16	Setting: Three military hospitals undergoing facility and staff integration Sample: 360 staff nurses on inpatient nursing units Type of intervention: Facilitated education and mentoring intervention Additional data in study: The Organizational Readiness for System-wide Integration of Evidence-Based Practice and EBP Implementation scale
Warren, 2016 [27]	Assessment name: Evidence-Based Practices (EBP) Beliefs scale Items mapped/items total: 16/16	Setting: 380-bed community teaching hospital and ambulatory care center (located in Maryland and part of a 10-hospital healthcare system) Sample: All RNs from the hospital (337 in 2008 and 342 in 2012) Type of intervention: Adoption of evidence-based practices by nurses Additional data in study: Evidence-based Practice Implementation scale (EBPI), and Organizational Culture & Readiness for System-wide Implementation of EBP scale (OCRSIEP)
OTHER ASSESSMENTS		
Aarons, 2004 [28]	Assessment name: Evidence-Based Practice Attitude Scale (EBPAS) Items mapped/items total: 15/15	Setting: 51 programs providing mental health services to children and adolescents and their families Sample: 322 public sector clinical service workers Type of intervention: EBPs Additional data in study: None
Anderson, 1998 [29]	Assessment name: Team Climate Inventory (TCI) Items mapped/items total: 61/61	Setting: British National Health Service Sample: 155 individuals from 27 hospital management teams; plus 121 groups in four occupations (35 primary health care teams, 42 social services teams, 20 psychiatric teams and 24 oil company teams, *N* = 971) Type of intervention: N/A Additional data in study: None
Armenakis, 2007 [30]	Assessment name: Organizational Change Recipients’ Beliefs Scale Items mapped/items total: 24/24	Setting: Developed iteratively in four studies Sample: Sample 1: 19 executives in an executive MBA program; Sample 2: 117 employees in the medical division of a not-for-profit; Sample 3: 117 employees at a U.S. durable goods manufacturer; Sample 4: 247 employees at a Public Safety Organization Type of intervention: various organizational changes depending on study Additional data in study: none
Bobiak, 2009 [31]	Assessment name: Measuring Practice Capacity for Change (MPCC) Items mapped/items total: 25/25	Setting: 3 health care systems in northeast Ohio Sample: 15 Primary Care Practices Type of intervention: EPOCHS (Enhancing Practice Outcomes through Communities and Health Systems) study, an ongoing group-randomized clinical trial to promote better quality management through the delivery of evidence-based health care Additional data in study: Direct observation and key informant interviews
Cherry, 2011 [32]	Assessment name: LTC Readiness Assessment Tool for EHR Implementation Items mapped/items total: 20/21	Setting: Long Term Care facilities in Texas Sample: 93 administrators Type of intervention: Electronic Health Record Additional data in study: None
Demiris, 2007 [33]	Assessment name: N/A Items mapped/items total: 20/22	Setting: Critical Access Hospitals in Missouri Sample: 27 administrators Type of intervention: Information and communication technology Additional data in study: None
Gibb, 2013 [34]	Assessment name: N/A Items mapped/items total: 20/20	Setting: Residential aged care facility Sample: 6 personal care workers Type of intervention: Evidence-based teamwork training system Additional data in study: Semi-structured interviews, semi-structured observation, focus groups
Gray, 2015 [11]	Assessment name: N/A Items mapped/items total: 53/55	Setting: Continuing care and rehabilitation facility in a major metropolitan city in Canada Sample: 194 staff Type of intervention: physical redevelopment and major shifts in operational and organizational processes Additional data in study: None
Helfrich, 2009 [35]	Assessment name: Organizational Readiness to Change Assessment (ORCA) Items mapped/items total: 74/74	Setting: 3 quality improvement projects in the US Veterans Health Administration Sample: 80 observations Type of intervention: various depending on project Additional data in study: Not reported
Nelson, 1999 [36]	Assessment name: Proactive Organizational Change: Assessing Critical Success Factors Items mapped/items total: 53/56	Setting: County Boards of Health Sample: 122 health board employees Type of intervention: Entering strategic partnership with non-profit hospital network Additional data in study: None
Pasmore, 1988 [37]	Assessment name: Sociotechnical Systems Assessment Survey (STSAS) Items mapped/items total: 87/100	**no use described in original publication, only measure itself**
Rubenstein, 2014 [38]	Assessment name: N/A Items mapped/items total: 90/90	Setting: 83 primary care practices in Minnesota Sample: 83 practices Type of intervention: Collaborative care model for improving depression care Additional data in study: Physician Practice Connection Questionnaire (modified to focus on depression care) and the Change Process Capability Questionnaire
Shaw, 2013 [39]	Assessment name: N/A Items mapped/items total: 22/22	Setting: US Veterans Health Administration Sample: 102 primary care physicians, non-physician providers, nurses, and information technology professionals Type of intervention: evidence-based nurse-delivered self-management phone intervention to facilitate hypertension management Additional data in study: Semi-structured interviews
Shea, 2014a [40]	Assessment name: N/A Items mapped/items total: 30/30	Setting: 47 ambulatory practices within an integrated delivery system Sample: 400 providers/staff Type of intervention: Meaningful Use of electronic health record Additional data in study: None
Shea, 2014b [41]	Assessment name: Organizational Readiness for Implementing Change (ORIC) Items mapped/items total: 12/12	Setting: Tested in 4 studies Sample: Study 1: 98 students at a university in southeastern U.S.; Study 2 and 3: 140 students at the same university; Study 4: 311 staff from international non-governmental organizations based in the U.S. Type of intervention: various depending on study Additional data in study: none
Toure, 2012a [42]	Assessment name: Practice Style Questionnaire Items mapped/items total: 17/17	Setting: Rehabilitation center in Montreal, Quebec, Canada Sample: 137 clinicians, 28 managers, and 47 nonclinical staff Type of intervention: e-Health Additional data in study: e-Health Readiness Measure
Toure, 2012b [42]	Assessment name: e-Health Readiness Measure Items mapped/items total: 57/57	Setting: Rehabilitation center in Montreal, Quebec, Canada Sample: 137 clinicians, 28 managers, and 47 nonclinical staff Type of intervention: e-Health Additional data in study: Practice Style Questionnaire
Zullig, 2013 [43]	Assessment name: N/A Items mapped/items total: 17/17	Setting: Academic hospital in Moshi, Tanzania Sample: 52 clinicians, nurses and administrators Type of intervention: Cancer registries Additional data in study: None

Assessments ranged in size from nine to 134 items (of note, both of these extremes were adaptations of the same original survey, discussed below), with a median of 30 items (interquartile range: 44 items). Whereas the first few assessments were larger, and large assessments with more than 50 items continue to be used, smaller assessments with fewer than 30 items, and often fewer than 20 items, began to appear in 2004. Overall these smaller assessments comprise roughly half of the assessment uses (52%, 15/29 uses).

The majority of uses represent separate assessments tailored to a unique context (62%, 18/29 uses), but three assessments were used more than once. Variations of the Texas Christian University Organizational Readiness for Change Treatment assessment were used six times. Originally developed for use in addiction treatment settings, this assessment includes both a director and a staff version [10]. All of the additional uses adapted or used supplemental data collection, and all but one use (in a child welfare setting) occurred in mental health/substance use settings. The second assessment to be used more than once was developed by Holt and colleagues, and was developed to better understand the use of information systems in a variety of organizations [8]. The second use of this assessment used the same questions in a primary care setting related to eHealth tool deployment, in addition to a supplemental questionnaire [24]. Finally, the Evidence-Based Practices Beliefs scale was used in three hospital-based settings, the first time when it was being developed [25] and later in two samples of hospital-based nurses as part of a battery of assessments [26, 27].

The 18 individually developed assessments were fielded in a variety of settings, including many types of clinical settings: primary care, long-term care, hospitals, rehabilitation, and mental health. Two assessments were not conducted in healthcare delivery settings [8, 30], but were included by Gagnon’s earlier work and had items that were deemed flexible enough to apply in healthcare delivery settings. All but six uses described a particular intervention for which the readiness assessment was conducted.

Eleven uses employed the specific phrase “Organizational Readiness for/to Change” [8, 10, 17, 21–24, 35, 41, 43], with an additional eight uses of “Organizational Readiness for [a specific intervention]” (e.g. e-Health) [11, 26, 27, 32, 38, 39, 42]. Other variations included “Practice Capacity for Change,” [31] “Preparedness for Change,” [34] and “Readiness to Engage in EBP.” [25]

### Items mapped to CFIR

**Fig. 2:**
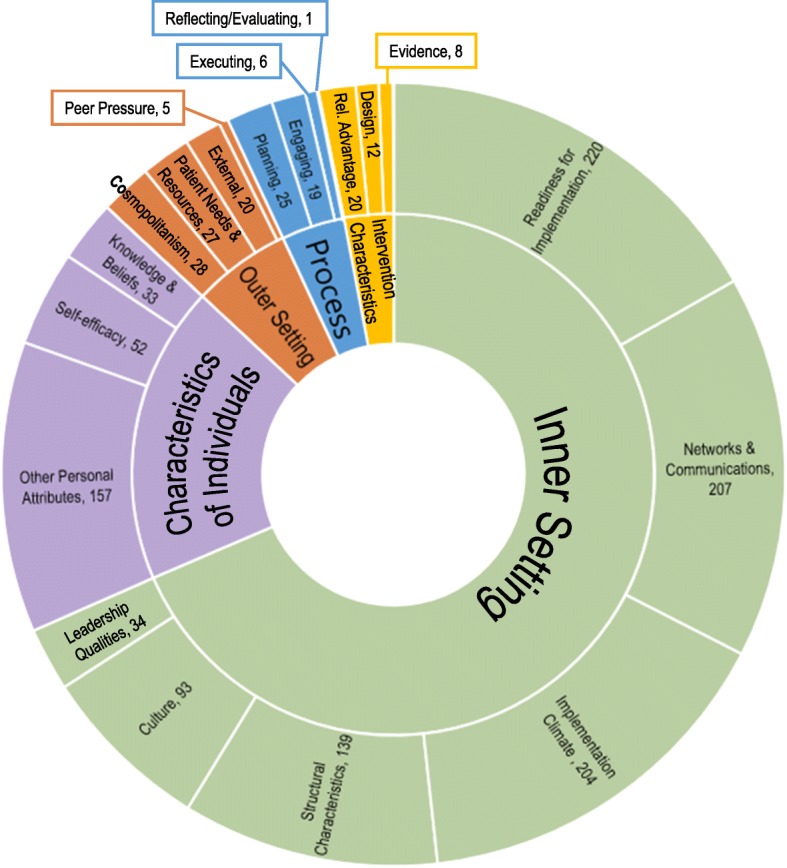
Items Mapped to CFIR

Of the 1370 readiness items, 1310 were mapped to CFIR; the remaining 60 items (4%) did not match with any of the CFIR constructs, largely due to the specificity of the question related to particular programming. The majority of items mapped to the CFIR domain of inner setting (68%, *n* = 897) (Fig. 2). The second most heavily mapped CFIR domain was characteristics of individuals (18%, *n* = 242), followed by outer setting (6%, *n* = 80), implementation process (4%, *n* = 51), and intervention characteristics (3%, *n* = 40).

Seven CFIR constructs had 50 or more items mapped to them, together accounting for 82% of the total items. Within the domain of inner setting, items most often mapped to the CFIR constructs of readiness for implementation (*n* = 220), networks and communications (*n* = 207), implementation climate (*n* = 204), structural characteristics (*n* = 139), and culture (*n* = 93). Within the domain of characteristics of individuals, items most often mapped to the CFIR constructs of other personal attributes (*n* = 157) and self-efficacy (*n* = 52).

We generated one additional construct-level code to capture a unique theme outside of the CFIR structure: leadership qualities (*n* = 34). These items did not fit into the CFIR categorizations, as the existing representation of leadership within CFIR was in sub-constructs related to engagement of leadership with a specific intervention, as opposed to a more general description of an organization’s leaders. See Fig. 2 for the mapping of items to CFIR domains and constructs.

### Assessments mapped to CFIR

**Fig. 3:**
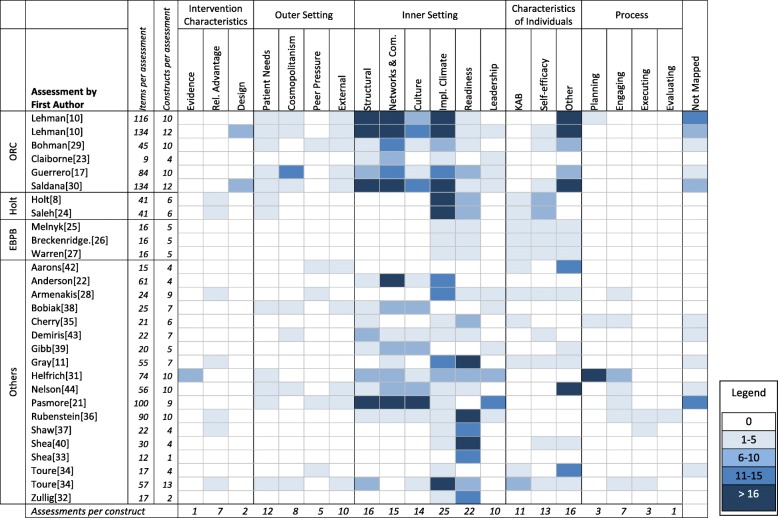
Heat Map of Assessment Uses to CFIR

Figure 3 is a heat map that displays the concentration of items within each assessment use across the various CFIR constructs and subconstructs. Twenty constructs had at least one item mapped from our bank of assessment items. The same seven CFIR constructs identified in the prior section as having most items mapped to them also occurred mostly frequently across assessment uses, however their order of popularity shifted: implementation climate was included in 25 uses; readiness for implementation was included in 22 uses; structural characteristics and personal attributes both included in 16 uses; networks and communications was included in 15 uses; culture was included in 14 uses; and self-efficacy was included in 13 uses. The number of constructs included in assessment uses ranged from one construct (readiness for implementation) in a 12-item assessment [41], to 13 constructs in a 57-item assessment [42]. The median number of constructs included in any assessment use was 6 constructs (interquartile range of 4 to 10), with median of 10 items per construct (interquartile range of 3 to 15).

### Top seven CFIR constructs

Highlighted here are the seven constructs that received the most attention from assessment developers and/or users, both in terms of the individual item analysis and the assessment heat map. Definitions and example items for all sub-constructs derived in the analysis are included in Additional file 3; here we focus on synthesizing findings for each construct.

#### Readiness for implementation

Readiness for implementation was mapped to the most individual items of any construct – 220 items – and ranked second in number of assessment uses, with 22 uses. Defined as “tangible and immediate indicators of organizational change,” readiness for implementation includes sub-constructs for leadership engagement, available resources, and access to knowledge and information [13]. CFIR defines this construct and its sub-constructs as specific to an intervention’s implementation, rather than describing the organization more generally. We identified 97 items as related to the subconstruct of available resources. The leadership engagement sub-construct was represented in 46 items, while access to knowledge and information about the intervention was represented by 13 items. The other 64 items in the readiness for implementation construct were judged to be “immediate indicators of organizational change” that fell outside available resources, leadership engagement, or access to knowledge and information [13]. These included items like “people who work here want to implement this change” [41], related to buy-in from staff members other than leadership, as well as items that described the meeting of pre-conditions for implementation (e.g., “how confident are you that most physicians can use e-prescribing instead of handwritten or printed prescriptions?” [40]).

#### Implementation climate

Implementation climate is defined as “the absorptive capacity for change, shared receptivity of involved individuals to an intervention and the extent to which use of that intervention will be rewarded, supported, and expected within their organization” [13]; in keeping with this definition, this set of codes was also specific to an intervention. This construct ranked first in assessment uses with 25 uses and third in total number of mapped items, with 204 items. Two main sub-constructs absorbed most of the items: compatibility (*n* = 97) and tension for change (*n* = 88). Compatibility of the intervention could be with the organization broadly, leadership, a workgroup or team, or the respondent’s beliefs or job. Items in the tension for change sub-construct took the form of needs assessments (e.g., “my unit needs guidance in developing services to address alcohol and drug behaviors presented by our patients” [21]), or descriptions of pressures for changes. The final 19 items in implementation climate were in the sub-constructs of relative priority (*n* = 7), organizational incentives and rewards (*n* = 6), goals and feedback (*n* = 4), and other (*n* = 2).

#### Other personal attributes

With 157 items, the other personal attributes construct ranked fourth in individual item mapping, and had 16 assessment uses. This broad construct within the characteristics of individuals domain served as catch-all for non-specific items about the respondent (e.g., self-efficacy or knowledge and beliefs about the intervention). Our inductive sorting produced seven sub-constructs, including a respondent’s engagement with or aptitude for learning new skills or job-related content (*n* = 51), descriptive information like identifying your position or department within the organization (*n* = 31), and whether respondents saw themselves as a self-described leader (*n* = 22).

#### Structural characteristics

Tied for third highest uses with other personal attributes and featured in slightly fewer individual items (*n* = 139), structural characteristics included five sub-constructs detailing various aspects of an organization’s workforce, physical and information technology infrastructure, and operational structure. These items were also not specific to any particular intervention.

#### Networks and communications

The networks and communications construct was also non-intervention specific, ranked second highest in individual items (*n* = 207), and had 15 assessment uses. We identified five sub-constructs related to relationships using definitions provided by the Lanham and colleagues model [18].

Mindfulness, which is defined as “openness to new ideas and different perspectives, fully engaged presence, rich discriminating awareness, or seeking novelty (even in routine situations)” [18], was represented by 91 items like “People in this team are always searching for fresh, new ways of looking at problems” [29]. Heedfulness is described as interactions “where individuals are sensitive to the task at hand (the job they are doing) and are paying attention to the way their roles and actions fit into (affect) the roles and actions of the entire group” [18]. “Everyone knows how their work will affect the work of the next person or the quality of the final product or service” [37] was an exemplar of items related to this sub-construct (*n* = 34). Respectful interaction items (*n* = 31), which represent “honest, self-confident, and appreciative interaction among individuals; often creating new meaning” [18], included this example: “Different parts of the organization work together well; when conflict arises, it is often productive” [37]. The Trust sub-construct, or the “willingness of an individual to be vulnerable to another individual” [18], contained 16 items, like “to what extent do you feel at ease with the members of your team?” [29]. The final sub-construct from the relationships model by Lanham and colleagues represented the idea of relatedness, which is “characterized by work- or non-work-related conversations and activities” [18]. Items in the relatedness sub-construct (*n* = 15) described communications like “staff share common goals about the care of residents at the beginning of and throughout each shift” [10].

In addition to the sub-constructs defined by the relationships model, two inductive sub-constructs were developed that captured communication activities that did not delve into underlying relationship traits: organization-level communications (*n* = 16), and cross-departmental communication (*n* = 4).

#### Self-efficacy

The construct for self-efficacy within the characteristics of individuals domain included 52 items; because they were consistent and coherent as a group and did not warrant subdivision, no further sub-constructs were developed.

#### Culture

The culture construct, which generally describes “norms, values, and basic assumptions of a given organization” [13] contained 93 items that were never specific to a particular implementation and described shared characteristics about the group/organization as a whole. This construct fell within the inner setting domain.

## Discussion

Our examination of organizational readiness for change assessments identified both significant variation and important commonalities in how scale developers operationalize this topic. Originally we had hoped to generate a master organizational readiness assessment with modular elements, in order to draw upon this assessment for our different projects. In reality, we found that the existing assessments were so tailored to the specific study, intervention, or setting that this was not possible. No gold standard exists within the realm of organizational readiness for change assessments; every use we identified was tailored to some degree, whether through modification or elimination of items from an existing assessment, supplemental data collection, or the building of an assessment de novo. And while the definition of organizational readiness for change can be either intervention-specific or more general, [7, 14, 44, 45] in developing measures authors chose to be intervention-specific for 23 of the 29 assessment uses we identified.

Use of the CFIR to compare content across assessments revealed several constructs that emerge frequently in readiness assessments, particularly implementation climate and readiness for implementation. These seem like core constructs, given their consistency in the data and conceptual overlap with the various definitions of organizational readiness for change. These constructs were never the only ones represented, however; every assessment also included a unique constellation of items across the other constructs and sub-constructs of CFIR. Structural characteristics, other personal attributes, and networks and communications constructs also appeared frequently, indicating the important role played by an organization’s structure, its people, and the connections between them in influencing readiness.

Using a broad framework like CFIR, rather than any specific organizational readiness for change framework, proved helpful because we were able to capture the full range of contextual information assessments were designed to gather, even when they did not directly overlap with one another. There were only minor adjustments needed to classify items from the assessments using CFIR, which suggests that organizational readiness for change is captured within the large framework of constructs “associated with effective implementation” that CFIR offers. The one notable area where we made additions to CFIR relates to teams. CFIR has domains for individual level (micro) and organizational level (macro) constructs, but no domain specific to an intermediate (meso) level, like a team. The prevalence of items we coded with the “team” unit of analysis suggests that this level may be distinct, but this distinction was somewhat obscured between the domain of inner setting, which often describes much more than one team, and the domain of characteristics of individuals, which is more granular. For instance, the wording of items like the following illustrate a team orientation: “The implementation team members have staff support and other resources required for the project” [35]. Capturing this team (meso) level as distinct from the macro level may be helpful in more clearly distinguishing the role of teams in readiness for change.

### Limitations

Several limitations may affect the interpretation of the presented findings. Potential publication bias must be acknowledged as an issue, but given that we were not focused on a particular outcome, we could not use standard statistical methods, like a funnel plot, for detecting potential bias. It is also likely that organizational readiness for change assessments, or assessments with the same purpose, are used either formally or informally in practice without being published. No unpublished assessments were included in this work, nor did we search gray literature for assessments, and as such, our conclusions may not be applicable to these additional assessments.

In addition, breaking down validated instruments into individual items can be problematic from the perspective of psychometric properties. However, our aim was not to produce valid instruments for use, but rather to conduct qualitative analysis, the aims of which are to describe themes and better understand commonalities and differences between assessments, rather than to test construct validity.

Finally, some items appear multiple times within our data set, which may impact our assessment of item and construct frequencies. In four cases these items came from identical assessments, which were tested in different populations. In addition, some adaptations included subsets of items from the original survey. Each use of a readiness assessment, regardless of duplication, was included to get a better understanding of our main objective, which was to describe the operationalization of organizational readiness for change as it appeared in the literature.

### Implications

A better understanding of organizational readiness for change may require the streamlining of terminology, but it is also important to describe why these assessments are done in the first place, and what developers felt was important to assess. While using a broad framework to identify commonalities suggests prevalent themes throughout the assessments, it is important we also understand how these constructs can be used to predict desired outcomes or serve as a diagnostic for tailoring implementation approaches. In addition, analysis of these assessments revealed an implicit expectation that team relationships are a critical component of readiness. There was no single construct within CFIR to capture this sentiment, but we found that Lanham and colleagues’ model for characteristics of work relationships was highly compatible with the content of relationship items. Future work more closely examining the role of relationships in organizational readiness may be required to fully explicate their impact.

## Conclusions

The readiness assessments reviewed in this article revealed significant commonalities; however, the specificity of many items suggests most assessments will need to be customized or tailored prior to use. The continued proliferation of new assessments, meanwhile, signals that there is no current gold standard assessment for organizational readiness for change. Consensus around a definition of organizational readiness for change may allow future developers to focus on a more parsimonious and better-specified set of constructs. Work testing the relationship between organizational readiness for change and implementation outcomes [46] will help to better specify the underlying mechanisms of readiness and may lead to more adaptable assessments. In the meantime, readiness assessments must often bridge the gap between measuring a theoretical construct and evaluating factors specific to a particular implementation.

## Supplementary information

**Additional file 1.** PRISMA Checklist.

**Additional file 2.** Search Strategy.

**Additional file 3.** Codebook.

**Additional file 4.** Coding Form.

## Data Availability

The datasets used and/or analyzed during the current study are available from the corresponding author on reasonable request. All literature included in the was obtained from publicly available sources.

## Abbreviations

CFIR: Consolidated framework for implementation research
HSR&D: Health services research and development
PRISMA: Preferred reporting items for systematic reviews and meta-analyses
QUERI: Quality enhancement research initiative
VA: Department of Veterans Affairs
