# Supplementary material for: Unpacking organizational readiness for change: an updated systematic review and content analysis of assessments
Source: BMC Health Serv Res. 2020 Feb 11;20:106. doi: 10.1186/s12913-020-4926-z (PMC7014613; doi:10.1186/s12913-020-4926-z)
Supplement: Supplementary file 2 — Additional file 2. Search Strategy. [file 12913_2020_4926_MOESM2_ESM.docx]

Additional file 2. Search Strategy

**ORGANIZATIONAL READINESS FOR CHANGE SEARCH METHODOLOGY**

**DATABASE SEARCHED & TIME PERIOD COVERED:**

PubMed – 1/1/2012-6/13/2017

**SEARCH STRATEGY:**

readiness[TIAB]

AND

Commitment[TIAB] OR Preparedness[TIAB] OR Acceptance[TIAB] OR Willingness[TIAB]

AND

Change[TI] OR Changing[TI] OR Organizational Innovation[MH] OR Organizational Innovation*[TIAB] OR Organisational Innovation*[TIAB] OR Organizational change*[TIAB] OR Organisational change*[TIAB] OR Institutional change*[TIAB] OR Institutional innovation*[TIAB] OR "stages of change"[TIAB]

AND

Organisation*[TIAB] OR Organization*[TIAB] OR Institutional*[TIAB] OR "Organization and Administration"[Mesh] OR "organization and administration" [Subheading] OR organizational innovation[Mesh]

======================================================================

**DATABASE SEARCHED & TIME PERIOD COVERED:**

Embase – 1/1/2012-6/13/2017

**SEARCH STRATEGY:**

readiness:ti,ab

AND

commitment:ab,ti OR preparedness:ab,ti OR acceptance:ab,ti OR willingness:ab,ti

AND

change:ti OR changing:ti OR 'organizational innovation':ab,ti OR 'organizational innovations':ab,ti OR 'organizational change':ab,ti OR 'organizational changes':ab,ti OR 'institutional change':ab,ti OR 'institutional changes':ab,ti OR 'institutional innovation':ab,ti OR 'institutional innovations':ab,ti OR

'stages of change':ab,ti

======================================================================

**DATABASE SEARCHED & TIME PERIOD COVERED:**

PsycINFO – 1/1/2012-6/13/2017

**SEARCH STRATEGY:**

TI readiness OR AB readiness

AND

TI ( Commitment OR Preparedness OR Acceptance OR Willingness ) OR AB ( Commitment OR Preparedness OR Acceptance OR Willingness )

AND

TI ( change OR Changing OR ‘‘Organizational Innovation’’ OR ‘‘Organisational Innovation’’ OR ‘‘Organizational change’’ OR ‘‘Organisational change’’ OR ‘‘Institutional change’’ OR ‘‘Institutional innovation’’ OR ‘‘Stages of change’’ ) OR AB ( change OR Changing OR ‘‘Organizational Innovation’’ OR ‘‘Organisational Innovation’’ OR ‘‘Organizational change’’ OR ‘‘Organisational change’’ OR ‘‘Institutional change’’ OR ‘‘Institutional innovation’’ OR ‘‘Stages of change’’) OR DE "Organizational Change"

AND

TI ( Organization* OR Organisation* OR Institutional* ) OR AB ( Organization* OR Organisation* OR Institutional* ) OR DE ( "organizational change" OR organizations )

LIMIT TO ACADEMIC JOURNALS

======================================================================

**DATABASE SEARCHED & TIME PERIOD COVERED:**

CINAHL – 1/1/2012-6/13/2017

**SEARCH STRATEGY:**

TI readiness OR AB readiness

AND

TI ( Commitment OR Preparedness OR Acceptance OR Willingness ) OR AB ( Commitment OR Preparedness OR Acceptance OR Willingness )

AND

TI ( change OR Changing OR ‘‘Organizational Innovation’’ OR ‘‘Organisational Innovation’’ OR ‘‘Organizational change’’ OR ‘‘Organisational change’’ OR ‘‘Institutional change’’ OR ‘‘Institutional innovation’’ OR ‘‘Stages of change’’ ) OR AB ( change OR Changing OR ‘‘Organizational Innovation’’ OR ‘‘Organisational Innovation’’ OR ‘‘Organizational change’’ OR ‘‘Organisational change’’ OR ‘‘Institutional change’’ OR ‘‘Institutional innovation’’ OR ‘‘Stages of change’’ ) OR DE "Organizational Change"

AND

TI ( Organization* OR Organisation* OR Institutional* ) OR AB ( Organization* OR Organisation* OR Institutional* ) OR ( "organizational change" OR organizations )

LIMIT TO PEER REVIEWED

======================================================================

**DATABASE SEARCHED & TIME PERIOD COVERED:**

Business Source Complete – 1/1/2012-6/13/2017

**SEARCH STRATEGY:**

TI readiness OR AB readiness

AND

TI ( Commitment OR Preparedness OR Acceptance OR Willingness ) OR AB ( Commitment OR Preparedness OR Acceptance OR Willingness )

AND

TI ( change OR Changing OR ‘‘Organizational Innovation’’ OR ‘‘Organisational Innovation’’ OR ‘‘Organizational change’’ OR ‘‘Organisational change’’ OR ‘‘Institutional change’’ OR ‘‘Institutional innovation’’ OR ‘‘Stages of change’’ ) OR AB ( change OR Changing OR ‘‘Organizational Innovation’’ OR ‘‘Organisational Innovation’’ OR ‘‘Organizational change’’ OR ‘‘Organisational change’’ OR ‘‘Institutional change’’ OR ‘‘Institutional innovation’’ OR ‘‘Stages of change’’ ) OR DE "Organizational Change"

AND

TI ( Organization* OR Organisation* OR Institutional* ) OR AB ( Organization* OR Organisation* OR Institutional* ) OR SU ( Organization OR "Organizational change" )

AND

SU ( health* OR medic* OR "Social service" OR "social services" ) OR TI ( health* OR medic* OR "Social service" OR "social services" ) OR AB ( health* OR medic* OR "Social service" OR "social services" )

LIMIT TO SCHOLARLY (PEER REVIEWED) JOURNALS

======================================================================

**DATABASE SEARCHED & TIME PERIOD COVERED:**

Web of Science Indexes=SCI-EXPANDED, SSCI, A&HCI, CPCI-S, CPCI-SSH, BKCI-S, BKCI-SSH, ESCI, CCR-EXPANDED, IC – 1/1/2012-6/13/2017

**SEARCH STRATEGY:**

ts=(readiness)

AND

ts=(Commitment OR Preparedness OR Acceptance OR Willingness)

AND

ts=(Change OR Changing OR Organizational Innovation OR Organizational Innovations OR Organisational Innovation OR Organisational Innovations OR Organizational change OR Organizational changes OR Organisational change OR Organisational changes ORInstitutional change OR Institutional changes OR Institutional innovation OR Stages of change)

AND

ts=(Organization* OR Organisation* OR Institutional*)

AND

ts=(Health* OR Medic* OR ‘‘Social service’’ OR "social services")

======================================================================

**DATABASE SEARCHED & TIME PERIOD COVERED:**

Sociological Abstracts – 1/1/2012-6/14/2017

**SEARCH STRATEGY:**

ab(readiness) OR ti(readiness) OR su(readiness) OR if(readiness)) OR (ab(Commitment OR Preparedness OR Acceptance OR Willingness) OR ti(Commitment OR Preparedness OR Acceptance OR Willingness) OR su(Commitment OR Preparedness OR Acceptance OR Willingness) OR if(Commitment OR Preparedness OR Acceptance OR Willingness)

AND

ti(change OR changing) OR ab("organizational innovation" OR "organizational innovations" OR "organizational change" OR "organizational changes" OR "institutional change" OR "institutional changes" OR "institutional innovation" OR "institutional innovations" OR "stages of change") OR ti("organizational innovation" OR "organizational innovations" OR "organizational change" OR "organizational changes" OR "institutional change" OR "institutional changes" OR "institutional innovation" OR "institutional innovations" OR "stages of change") OR su("organizational innovation" OR "organizational innovations" OR "organizational change" OR "organizational changes" OR "institutional change" OR "institutional changes" OR "institutional innovation" OR "institutional innovations" OR "stages of change") OR if("organizational innovation" OR "organizational innovations" OR "organizational change" OR "organizational changes" OR "institutional change" OR "institutional changes" OR "institutional innovation" OR "institutional innovations" OR "stages of change")

AND

ab(health* OR medic* OR "social service" OR "social services") OR ti(health* OR medic* OR "social service" OR "social services") OR su(health* OR medic* OR "social service" OR "social services") OR if(health* OR medic* OR "social service" OR "social services")

AND

ab(organization* OR organisation* OR institutional*) OR ti(organization* OR organisation* OR institutional*) OR su(organization* OR organisation* OR institutional*) OR if(organization* OR organisation* OR institutional*)
