# Supplementary material for: Unpacking organizational readiness for change: an updated systematic review and content analysis of assessments
Source: BMC Health Serv Res. 2020 Feb 11;20:106. doi: 10.1186/s12913-020-4926-z (PMC7014613; doi:10.1186/s12913-020-4926-z)
Supplement: Supplementary file 3 — Additional file 3. Codebook. [file 12913_2020_4926_MOESM3_ESM.docx]

Additional file 3. CFIR Codebook

The following codebook is organized by CFIR’s structure: each of the five domains and the corresponding constructs and sub-constructs are listed below with example items from the item bank. Additional information about the coding process is noted in the right-hand columns.

| 1. **INTERVENTION CHARACTERISTICS** | Specific to a particular intervention? | Notes |
| --- | --- | --- |
| 1. **Intervention Source:** *Perception of key stakeholders about whether the intervention is externally or internally developed*. | ✓ | Not found in study data set |
| 1. **Evidence Strength & Quality**: *Stakeholders’ perceptions of the quality and validity of evidence supporting the belief that the intervention will have desired outcomes.* Example: “The {proposed practice changes or guideline implementation} are supported by clinical experience with VA patients” Helfrich, 2009{\ | ✓ |  |
| 1. **Relative Advantage**: *Stakeholders’ perception of the advantage of implementing the intervention versus an alternative solution*. Example: “The new process of care for depression is more advantageous than the old to everyone involved (patients, personnel, and medical group)” Rubenstein, 2014 | ✓ |  |
| 1. **Adaptability**: *The degree to which an intervention can be adapted, tailored, refined, or reinvented to meet local needs.* | ✓ | Not found in study data set |
| 1. **Trialability**: *The ability to test the intervention on a small scale in the organization [8], and to be able to reverse course (undo implementation) if warranted.* | ✓ | Not found in study data set |
| 1. **Complexity**: *Perceived difficulty of implementation, reflected by duration, scope, radicalness, disruptiveness, centrality, and intricacy and number of steps required to implement* | ✓ | Not found in study data set |
| 1. **Design Quality and Packaging**: *Perceived excellence in how the intervention is bundled, presented, and assembled.* Example: “The instruction methods for learning new counseling strategies or materials that work best for you are: self-study” Lehman, 2002b | ✓ |  |
| 1. **Cost**: *Costs of the intervention and costs associated with implementing that intervention including investment, supply, and opportunity costs*. | ✓ | Not found in study data set |
| 1. **OUTER SETTING** | Specific to a particular intervention? | Notes |
| 1. **Patient Needs & Resources**: *The extent to which patient needs, as well as barriers and facilitators to meet those needs are accurately known and prioritized by the organization.* Example: “When we adopt this change, we will be better equipped to meet our customers' needs.” Holt, 2007 |  |  |
| 1. **Cosmopolitanism**: *The degree to which an organization is networked with other external organizations.* Example: “How well does your agency work collaboratively with programs that provide public health services?” Guerrero, 2016 |  |  |
| 1. **Peer Pressure**: *Mimetic or competitive pressure to implement an intervention; typically because most or other key peer or competing organizations have already implemented or in a bid for a competitive edge.* Example: “It is best to change the way I treat a certain problem when my peers are making the same changes.” Toure, 2012a |  |  |
| 1. **External Policy & Incentives**: *A broad construct that includes external strategies to spread interventions including policy and regulations (governmental or other central entity), external mandates, recommendations and guidelines, pay-for-performance, collaboratives, and public or benchmark reporting.* Example: “Current pressures to make program changes come from accreditation or licensing authorities.” Lehman, 2002b |  |  |
| 1. **INNER SETTING**   (Examples provided only for constructs represented in the study sample) | Specific to a particular intervention? | Notes |
| 1. **Structural Characteristics**: *The social architecture, age, maturity, and size of an organization.*   Example: “There are not enough staff in my workplace to provide quality care.” Gibb, 2013   1. **IT Infrastructure**: *The nature, quality, or character of the information technology infrastructure of an organization.* Example: “How many laptop computers does your institution have? None/1-3/4-6/7 or more?” Demiris, 2007 2. **Performance Evaluation**: *The nature, quality, or character of performance evaluation processes of an organization.* Example: “Many people would say that they have influence on the performance ratings their peers receive.” Pasmore, 1988 3. **Physical Infrastructure**: *The nature, quality, or character of the physical infrastructure of an organization.* Example: “Your offices and equipment are adequate.” Lehman, 2002b 4. **Policy and Procedures**: *The nature, quality, or character of the general policies or procedures of an organization.* Example: “Agency policy (rules and procedures): always facilitates operations/sometimes facilitates operations/impedes operations.” Nelson, 1999 5. **Workforce**: *The nature, quality, or character of the workforce at an organization.* Example: “There are not enough staff in my workplace to provide quality care.” Gibb, 2013 |  | Sub-constructs 1-5 created by study team inductively |
| 1. **Networks & Communications**: *The nature and quality of webs of social networks and the nature and quality of formal and informal communications within an organization.* Example: “Nonclinical staff often receive incompatible requests from 2 or more people.” Bobiak, 2009 2. **Trust**: *Willingness of an individual to be vulnerable to another individual.* Example: “To what extent do you feel at ease with the members of your team?” Anderson, 1998 3. **Mindfulness**: *(1) Openness to new ideas and different perspectives, (2) fully engaged presence, (3) rich discriminating awareness, or (4) seeking novelty (even in routine situations).* Example: “People in this team are always searching for fresh, new ways of looking at problems.” Anderson, 1998 4. **Heedfulness**: *Interaction where individuals are sensitive to the task at hand (the job they are doing) and are paying attention to the way their roles and actions fit into (affect) the roles and actions of the entire group.* Example: “Everyone knows how their work will affect the work of the next person or the quality of the final product or service.” Pasmore, 1988 5. **Respectful Interaction**: *Honest, self-confident, and appreciative interaction among individuals; often creating new meaning.* Example: “Different parts of the organization work together well; when conflict arises, it is often productive.” Pasmore, 1988 6. **Relatedness**: *Characterized by work- or non-work-related conversations and activities.* Example: “Staff share common goals about the care of residents at the beginning of and throughout each shift.” Lehman, 2002b 7. **Org Level Communication**: *The nature, quality, or character of the general organization-wide communication.* Example: “The formal and informal communication channels here work very well.” Lehman, 2002b 8. **Cross Department Communication**: *The nature, quality, or character of the cross-departmental communication within an organization.* Example: “Senior leadership/clinical management in {your organization} promote communication among clinical services and units.” Helfrich, 2009 |  | Sub-constructs 1-5 from Lanham, et al, 2009;  6-7 created by study team inductively |
| 1. **Culture**: *Norms, values, and basic assumptions of a given organization.* 2. **Shared Values**: *General values shared by members of an organization.* Example: “This practice has a shared vision among practice members.” Bobiak, 2009 3. **Prevalent Sentiments**: *General attitudes or emotions shared by members of an organization.* Example: “Staff members often show signs of stress and strain.” Lehman, 2002b 4. **Learning Culture**: *Evidence of norms, values, assumptions, practices, or processes that encourage the development of knowledge and competence that is not related to the specific intervention.* Example: “This program encourages and supports professional growth.” Lehman, 2002b |  | Sub-constructs 1-3 created by study team inductively |
| 1. **Implementation Climate**: *The absorptive capacity for change, shared receptivity of involved individuals to an intervention and the extent to which use of that intervention will be rewarded, supported, and expected within their organization.* Example: “We have the capability to successfully implement the change.” Armenakis, 2007 2. **Tension for Change**: *The degree to which stakeholders perceive the current situation as intolerable or needing change.* Example: “Current pressures to make program changes come from program supervisors or managers.” Lehman, 2002b 3. **Compatibility**: *The degree of tangible fit between meaning and values attached to the intervention by involved individuals, how those align with individuals’ own norms, values, and perceived risks and needs, and how the intervention fits with existing workflows and systems.* Example: “I think we are spending a lot of time on changes related to the move to the new hospital when the senior managers don’t even want it implemented.” Gray, 2015 4. **Relative Priority**: *Individuals’ shared perception of the importance of the implementation within the organization.* Example: “How much does your facility want to implement this change?” Zullig, 2013 5. **Organizational Incentives & Rewards**: *Extrinsic incentives such as goal-sharing awards, performance reviews, promotions, and raises in salary and less tangible incentives such as increased stature or respect.* Example: “When we implement this change, i can envision financial benefits coming my way.” Holt, 2007 6. **Goals and Feedback**: *The degree to which goals are clearly communicated, acted upon, and fed back to staff and alignment of that feedback with goals.* Example: “Senior leadership/clinical management/staff opinion leaders agree on the goals for this intervention.” Helfrich, 2009 7. **Learning Climate**: *A climate in which: a) leaders express their own fallibility and need for team members’ assistance and input; b) team members feel that they are essential, valued, and knowledgeable partners in the change process; c) individuals feel psychologically safe to try new methods; and d) there is sufficient time and space for reflective thinking and evaluation.* | ✓ | Sub-construct 6 not found in study data set |
| 1. **Readiness for Implementation**: *Tangible and immediate indicators of organizational commitment to its decision to implement an intervention.* 2. **Leadership Engagement**: *Commitment, involvement, and accountability of leaders and managers with the implementation.* Example: “My immediate manager encourages me to support this change.” Armenakis, 2007 3. **Available Resources**: *The level of resources dedicated for implementation and on-going operations including money, training, education, physical space, and time.* Example: “Our resources (personnel, time, financial) are too tightly limited to improve depression care” Rubenstein, 2014 4. **Access to Knowledge and Information**: *Ease of access to digestible information and knowledge about the intervention and how to incorporate it into work tasks.* Example: “The availability of information about what is happening within your agency: adequate/increasing/inadequate” Nelson, 1999 5. **Other**:  *Other tangible and immediate indicators of organizational commitment to its decision to implement an intervention.* Example: “how confident are you that most physicians can use e-prescribing instead of handwritten or printed prescriptions?” Shea, 2014a | ✓ |  |
| 1. **Leadership Qualities**: *Descriptions of organization leaders or management that are not specific to the intervention or its implementation.* Example: “Senior Leadership/clinical management in {your organization} hold staff members accountable for achieving results.” Helfrich, 2009 |  | Construct created by study team inductively |
| 1. **CHARACTERISTICS OF INDIVIDUALS**   (Examples provided only for constructs represented in the study sample) | Specific to a particular intervention? | Notes |
| 1. **Knowledge & Beliefs about the Intervention**: *Individuals’ attitudes toward and value placed on the intervention as well as familiarity with facts, truths, and principles related to the intervention.* Example: “I am sure that evidence-based guidelines can improve clinical care.” Melnyk, 2008 | ✓ |  |
| 1. **Self-efficacy**: *Individual belief in their own capabilities to execute courses of action to achieve implementation goals.* Example: “How confident are you that you can use e-prescribing instead of handwritten or printed prescriptions?” Shea, 2014a | ✓ |  |
| 1. **Individual Stage of Change**: *Characterization of the phase an individual is in, as he or she progresses toward skilled, enthusiastic, and sustained use of the intervention.* | ✓ | Not found in study data set |
| 1. **Individual Identification with Organization**: *A broad construct related to how individuals perceive the organization and their relationship and degree of commitment with that organization.* |  | Not found in study data set |
| 1. **Other Personal Attributes**: *A broad construct to include other personal traits such as tolerance of ambiguity, intellectual ability, motivation, values, competence, capacity, and learning style.* 2. **Busy**: *The degree to which an individual is overwhelmed with time commitments or has too much to do.* Example: “I will not have the time to read up on every practice decision.” Toure, 2012a 3. **Descriptive Information**: *Various demographic facts or descriptive information about an individual’s role.* Example: “What percent of your time do you spend performing clinic service provision activities?” Nelson, 1999 4. **Experience vs. Evidence-based Practice**: *The degree to which an individual values clinical experience over evidence for clinical decision-making.* Example: “I know better than academic researchers how to care for my clients.” Aarons, 2004 5. **Learning Capacity**: *The degree to which an individual is willing and able to learn.* Example: “Learning and using new procedures are easy for you.” Lehman, 2002a 6. **Personality**: *Descriptions of an individual’s personality traits, aside from those that are specifically a part of other sub-constructs in this section.* Example: “My colleagues consider me to be someone who marches to the beat of my own drum (does my own thing).” Toure, 2012a 7. **Self-Described Leader**: *The degree to which an individual perceives themselves to be a leader.* Example: “You are considered an experienced source of advice about services.” Guerrero, 2016 8. **Miscellaneous**: *Other various personal attributes of individuals not included in other sub-constructs in this section.* Example: “You have the skills to conduct effective staff meetings.” Lehman, 2002b |  | Sub-constructs 1-7 created by study team inductively |
| 1. **PROCESS**   (Examples provided only for constructs represented in the study sample) | Specific to a particular intervention? | Notes |
| 1. **Planning**: *The degree to which a scheme or method of behavior and tasks for implementing an intervention are developed in advance and the quality of those schemes or methods.* Example: “The implementation plan for this intervention identifies specific roles and responsibilities.” Helfrich, 2009 | ✓ |  |
| 1. **Engaging**: *Attracting and involving appropriate individuals in the implementation and use of the intervention through a combined strategy of social marketing, education, role modeling, training, and other similar activities.* 2. **Opinion Leaders**: *Individuals in an organization who have formal or informal influence on the attitudes and beliefs of their colleagues with respect to implementing the intervention.* Example: “The majority of my respected peers are dedicated to making this change successful.” Armenakis, 2007 3. **Formally Appointed Internal Implementation Leaders**: *Individuals from within the organization who have been formally appointed with responsibility for implementing an intervention as coordinator, project manager, team leader, or other similar role.* Example: “The leaders of our efforts to improve depression care are enthusiastic about their task.” Rubenstein, 2014 4. **Champions**: *Individuals who dedicate themselves to supporting, marketing, and ‘driving through’ an [implementation]” , overcoming indifference or resistance that the intervention may provoke in an organization.* Example: “The project clinical champion accepts responsibility for the success of this project.” Helfrich, 2009 5. **External Change Agents**: *Individuals who are affiliated with an outside entity who formally influence or facilitate intervention decisions in a desirable direction.* Example: “Outreach to and involvement of external stakeholders in the planning process: comprehensive involvement in all phases of planning/some collaboration with other agencies/little input” Nelson, 1999 | ✓ |  |
| 1. **Executing**: *Carrying out or accomplishing the implementation according to plan.*   Example: “How confident are you that the core implementation group can effectively coordinate the efforts of those involved in implementing this program?” Shaw, 2013 | ✓ |  |
| 1. **Reflecting & Evaluating**: *Quantitative and qualitative feedback about the progress and quality of implementation accompanied with regular personal and team debriefing about progress and experience.* Example: “We have greatly improved the process of depression care in the past year.” Rubenstein, 2014 | ✓ |  |
