# Supplementary material for: Unpacking organizational readiness for change: an updated systematic review and content analysis of assessments
Source: BMC Health Serv Res. 2020 Feb 11;20:106. doi: 10.1186/s12913-020-4926-z (PMC7014613; doi:10.1186/s12913-020-4926-z)
Supplement: Supplementary file 4 — Additional file 4. Coding Form. [file 12913_2020_4926_MOESM4_ESM.docx]

Additional file 4. Readiness Items Coding Form

Below is the form that was populated for every item within the item bank. Readiness assessment, item number, and item text were pre-populated. Coders chose a CFIR construct from the codebook (Appendix B), and also completed other coding form fields to describe the unit of measurement (i.e., the item’s subject, when unclear the organization level served as the default), whether the item referred to a specific intervention or was more general in phrasing, and whether the item was phrased in a negative way.

Readiness Assessment Name/Author ___________________________________ Item Number _______

Readiness Assessment Item Text: _________________________________________________________

CFIR Construct: ________________________________________________________________________

Unit of Measurement:

- Organization
- Leadership
- Team
- Staff
- Self
- Specific to intervention?
- Negative valence
- Flag for Discussion

Notes: _______________________________________________________________________________
